# Sparse Regularization-Based Approach for Point Cloud Denoising and Sharp Features Enhancement

**DOI:** 10.3390/s20113206

**Published:** 2020-06-05

**Authors:** Esmeide Leal, German Sanchez-Torres, John W. Branch

**Affiliations:** 1 Faculty of Engineering, Universidad Autónoma del Caribe, Barranquilla 080001, Colombia; esleal@uac.edu.co; 2 Faculty of Engineering, Universidad del Magdalena, Santa Marta 470004, Colombia; 3 Facultad de Minas, Universidad Nacional de Colombia, Sede Medellín 050041, Colombia; jwbranch@unal.edu.co

**Keywords:** point cloud denoising, 3D surface reconstruction, sparse representation

## Abstract

Denoising the point cloud is fundamental for reconstructing high quality surfaces with details in order to eliminate noise and outliers in the 3D scanning process. The challenges for a denoising algorithm are noise reduction and sharp features preservation. In this paper, we present a new model to reconstruct and smooth point clouds that combine L1-median filtering with sparse L1 regularization for both denoising the normal vectors and updating the position of the points to preserve sharp features in the point cloud. The L1-median filter is robust to outliers and noise compared to the mean. The L1 norm is a way to measure the sparsity of a solution, and applying an L1 optimization to the point cloud can measure the sparsity of sharp features, producing clean point set surfaces with sharp features. We optimize the L1 minimization problem by using the proximal gradient descent algorithm. Experimental results show that our approach is comparable to the state-of-the-art methods, as it filters out 3D models with a high level of noise, but keeps their geometric features.

## 1. Introduction

With the rapid expansion of 3D scanning devices, the process of capturing and scanning real objects has become a common task in many areas, ranging from medicine and entertainment to industry and 3D printing. Despite significant development in the precision of 3D scanning technology, the raw data produced by the scanning devices inevitably contains noise and outliers caused by the inherent measurement error of 3D devices and the digitalization process. Herein lies the importance of denoising the point cloud in a pre-processing step before proceeding with surface reconstruction or shape analysis. The goal of the denoising algorithms is to suppress noise and outliers while preserving the sharp features such as edges and corners. Unlike denoising methods focused on triangular meshes, methods focused on point clouds do not have connectivity information, introducing an additional challenge. Denoising point clouds with sharp features is a complex problem as the features and noise are high frequency and therefore difficult to distinguish.

Many point set surfaces are piecewise smooth almost everywhere except for a few features such as corners and edges [1,2]. This means that these features are sparse, allowing a sparsity analysis in the point clouds to be conducted to estimate them. We can measure the sparsity of a solution using either the L0 norm or the L1 norm. The L0 norm counts the number of non-zero elements in a vector, directly measuring the sparsity, but it is challenging to optimize due to its non-convexity; furthermore, the L1 norm can approximate the L0 norm. The L1 norm is convex, and under certain conditions, produces sparse solutions. Some works exploit the sparsity theory on point clouds [1,2,3,4], and applying this theory is motivated by the field of sparse signal reconstruction and compressed sensing [5,6]. These works have attempted to overcome the problems related to noisy point clouds; the algorithms proposed in these studies perform well for feature preservation with a certain level of noise. Still, when the noise scale is larger or impulsive noise is present, they usually do not do well. Although the L0 norm produces sparser solutions than the L1 norm, the application of the L0 minimization can over-flatten and over-sharpened effects for small geometric features.

In this paper, we propose a robust method that focuses on removing noise and outliers while preserving the sharp features in a point cloud. Our approach comprises two iterative steps: (1) the normal estimation, finding a regression plane equidistant to all heights in a local neighborhood to then calculate the normal at the plane; and (2) based on the estimation of the normals, the position of the points is updated, using the orthogonal distance of the noisy point to the local regression plane, shifting the point along the normal direction projecting it onto the plane. This two-step procedure is repeated until a minimum error threshold is reached.

Our work is motivated by three observations: (1) L1-median filtering data-fidelity term encourages us to find a local regression plane to approximate the input points while discarding the noise and outliers; (2) points that belong to the same local smooth region will have similar normals, and the differences between them should be sparse, while large differences in values would reveal sharp features; and (3) points in a local neighborhood must comply with a local planarity criterion, except in the sharp features.

The three principal contributions of this paper are as follows: We present a two-step method for both normal estimation and point position update measuring the sparsity of sharp features while discriminating between noise and features. First, an adaptive weighted strategy is used to improve the normal estimation on sharp features. Second, a point cloud denoising method is developed that is sufficiently robust to large scale noise, outliers, and impulsive noise, outperforming some state-of-the-art point clouds denoising methods.

## 2. Related Work

Point cloud denoising algorithms can be roughly divided into six categories as follows: moving least squares (MLS)-based methods, locally optimal projection (LOP)-based methods, sparsity-based methods, non-local similarity-based methods, graph-based methods, and normal smoothing-based methods. In this section, we are interested in point cloud denoising coupled with features preservation.

### 2.1. MLS-Based Methods

The MLS [7] methods approximate a noisy input point cloud with a smooth surface by projecting the noisy points onto the MLS surface. Three steps are required to project each point: (1) finding a local reference domain to each point, (2) defining a function above the reference domain by fitting a bivariate polynomial using its neighboring points, and (3) computing the projection by evaluating the polynomial at the origin. MLS methods have some drawbacks, because they are not robust to outliers. The projection procedure can be unstable in high curvature regions and for low sampling rate and can over-smooth the surface. Several variants to this method have been proposed to correct the cited problems and for handling sharp features; e.g., algebraic point set surfaces (APSS) [8], and robust implicit MLS (RIMLS) [9].

### 2.2. LOP-Based Methods

Without the use of normal information, LOP-based [10] methods generate a set of points called particles using the L1 median and a regularization term. This method projects the points onto an underlying surface while enforcing a uniform distribution of points. A variation to this method is the weighted LOP (WLOP) [11]; this method produces more evenly distributed points on the surface. Edge aware resampling (EAR) [12] improves sharp features by modifying LOP to use normal information as a weight function. However, LOP-based methods can produce over-smoothing because of the use of local operators.

### 2.3. Non-Local Similarity-Based Methods

These methods are inspired by the image processing techniques non-local mean (NLM) [13] and the block-matching and 3D filtering (BM3D) [14] algorithms, and they exploit the concepts of self-similarity between small surface patches in the point cloud. The direct application of this concept to the point cloud is not straightforward, because the point cloud structure does not exhibit a regular disposition such as the pixels in an image in their structure. The methods based on MLN or BM3D better preserve structural features under a high level of noise. One of the first works extending the NLM algorithm to operate in point clouds is [15]. This method uses the MLS surface and its polynomial coefficients as neighborhood descriptors to find similar patches or neighborhoods. In [16], the authors generalized the BM3D, searching for similar neighborhoods globally in the point cloud using the iterative closest point (ICP) algorithm. Low-rank matrix representation is used in [4], wherein the authors use dictionary representation from the noisy patches to smooth 3D patches. The drawback of this method is its computational complexity given the global point clouds search.

### 2.4. Graph-Based Methods

Graph-based methods treat the point cloud as a signal in a graph, and the smooth surface is chosen by using graph filters. In [17], the authors use the graph of the k-nearest neighborhood to represent a point cloud as a signal, and carry the smoothing out via convex optimization. The method in [18] used weighted graph Laplacian over the normals and total variation L1-norm as the regularized term to model two kinds of additive noise. Using a bipartite graph, they establish a linear relationship between the points and normals to proceed with the optimization. In [19], the authors proposed the use of a graph Laplacian regularization (GLR) and low manifold model to find self-similarity between patches to the smooth point cloud, avoiding the direct smoothing of point coordinates or point normals. In [20], the authors computed local tangent planes based on a graph and then reconstructed the point cloud by the weighted average of its projections in the tangent planes.

### 2.5. Normal Smoothing-Based Methods

Normal smoothing methods are focused on estimating noise-free normals, followed by an updating of the position of the points based on the clean normals. In [21], the authors used a robust version of principal component analysis (PCA) to estimate the normal vector; the authors proposed weight factors that were inversely proportional to the sum of the distance to the mean. The weights defined in this way make the method robust to outliers and noise. They proposed a simple solution to avoid data shrinkage using bootstrap bias correction. The method iteratively smooths the surface and preserves sharp features.

In [22], the authors used the multi-normal guided concept (GN) to correctly estimate the normals in edges and corners, based on the observation that points on the same side of the edges have the same normal orientation. The first step is to detect the edges and then refine them using the L1-medial skeleton of point cloud algorithm [23], followed by an estimation of the multi-normals, and finally, an updating of the position by optimizing a height-based function. In [24], the authors used the same multi-normal concept for denoising called rolling normal filtering (RN). The first step here was the same as for [22], but for the point position update, they introduced an energy term to avoid point cloud deformation close to the edges. The method is robust to the noise and preserves edges and corners. Yadav et al. [25] used Constraint-based normal voting tensor (CVN) analysis and binary optimization to estimate noise-free normal. Next, to update the position of points, the method was used to classify each point on the cloud as edge, corner, or planar point, and based on this classification, they proposed three optimization procedures for each type of point. The method is effective for denoising the point cloud and preserves the sharp features.

### 2.6. Sparsity-Based Methods

These methods are based on the theory of sparse representation of certain geometric features of the point cloud. The sparsity-based method assumes local planarity for the optimization model. Recently, attention has been devoted to sparsity-based methods in geometry processing [26]. These methods comprise two sparse modeling steps. The first carries out a sparse reconstruction of the normals by solving a global minimization problem with sparse prior regularization. The prior model can be the L0-norm or L1-norm. The local planarity comprises the following assumption: If two points belong to the same smooth region, its normal vectors will be similar; therefore, the gradient should be sparse. Based on the smoothed normals, it updates the point positions following a second sparse global model optimization. Methods in [1,2] follow this strategy. Recently, authors in [3] proposed a method called moving robust principal component analysis (RMPCA), using weighted minimization of the point deviations from a local reference plane to preserve sharp features. However, when the noise level is high, over-smoothing or over-sharpening occurs. Our approximation belongs to sparsity-based methods and is in the same spirit of RMPCA, but the difference lies in that we use sparsity in both data fitting and the prior term. Our method uses the L1 median for the fitting term and the L1 norm for the regularization term. For the proposed method, we apply a local sparse optimization strategy based on proximal gradient.

## 3. Robust Point Cloud Denoising

As in previous point cloud denoising methods [22,24,25], our method comprises two steps, namely normal denoising and point position update.

**Figure 1:**
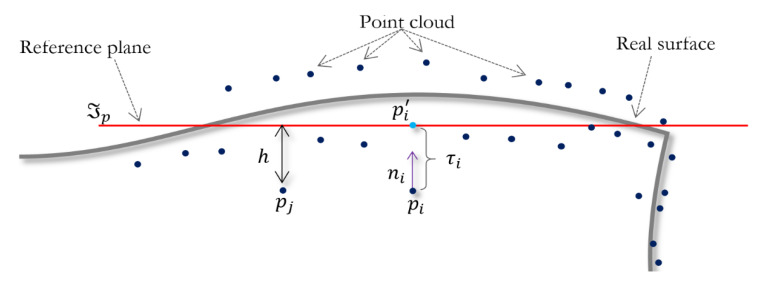
The point $p_{i}$ is projected onto the reference plane. ${ℑ}_{p}$ is a linear approximation to the surface.

Starting from a noisy point cloud P near an unknown surface S, the goal of the proposed algorithm is to find a noise-free point set $P^{\prime }$ that conserves the features of the original point cloud. We use local neighborhoods to each point $p_{i}\in P$. The main idea is to define a local reference tangent plane ${ℑ}_{p}$ to every $p_{i}$ in the point cloud and determine its normal vector $n_{i}$, and then shift the point $p_{i}$ in the normal direction to a distance $\tau_{i}\in \mathbb{R}$, obtaining a new position ${p^{\prime }}_{i}=p_{i}+\tau_{i}n_{i}$, ${p^{\prime }}_{i}\in P^{\prime }$. The new position ${p^{\prime }}_{i}$ being the projection of point $p_{i}$ onto the tangent plane ${ℑ}_{p}$, which is the linear approximation of the surface S at point $p_{i}$, as shown in Figure 1.

The normal $n_{i}$ and the displacement $\tau_{i}$, are computed iteratively by adjusting the tangent plane ${ℑ}_{p}$ to the neighborhood $N_{g}\left(p_{i}\right)$. To estimate the tangent plane ${ℑ}_{p}$, we are looking for an equidistance height to all heights of the points $p_{j}\in N_{g}\left(p_{i}\right)$ over ${ℑ}_{p}$. To estimate the local reference tangent plane ${ℑ}_{p}$, we minimize a cost function (Equation (2)) concerning τ and n, subject to the constraint ‖n‖=1.

### 3.1. L1-Median

The L1-median is a robust estimator related to the multivariate median, and is defined to be the point p, which minimizes the sum of Euclidean distances to all points in the data set ${\left\{p_{j}\right\}}_{j\in J}$.

(1) $$\arg\;\underset{x}{\min}\underset{j\in J}{\sum }\|p_{j}-p\|$$

The L1-median is insensitive to outliers and noise when compared with the mean [10]. We use the L1-median as a data-fidelity term.

### 3.2. L1 Sparse Regularization

L1 regularization has been applied for feature selection [27], sparse signal reconstruction [28], signal processing as image decomposition [29], and basis pursuit [30]. Although L0 regularization produces the sparsest solution, under certain constraints, L1 regularization produces a sparse solution [31]. Image processing has successfully applied L1 regularization to preserve fine details and edges through the minimization of the gradient [32]. This is conceptually named total-variation regularization or TV, and is used to measure the sparsity of the gradient. The proposed method uses TV for normal estimation to preserve the sharp features.

### 3.3. Cost Function

To denoise the noisy point cloud, we integrate L1-median height filter and L1 regularization of gradient or total variation. The normal is obtained, minimizing the following energy functional:

(2) $$\underset{n,\tau }{\min}E_{f}+\lambda E_{reg}$$

where $E_{f}$ is the fidelity term, $E_{reg}$ is the regularization term, λ is the regularization parameter, and ‖n‖=1. 

The L1-median height fidelity term $E_{f}$ is defined as follows:

(3) $$E_{f}=\underset{q_{j}\in N_{g}\left(p_{i}\right)}{\sum }\|h_{i}-\tau_{i}\|\psi \left(h_{i},\tau_{i}\right)\theta \left(\left\|p_{i}-q_{j}\right\|\right)$$

where $h_{i}=n_{i}^{t}\left(p_{i}-q_{j}\right)$, $\psi \left(h_{i},\tau_{i}\right)=e^{-{\left(h_{i}-\tau_{i}\right)}^{2}/\sigma_{h}^{2}}$, and $\theta \left(\left\|p_{i}-q_{j}\right\|\right)=e^{-d^{2}/\sigma_{d}^{2}}$.

It is used to fit a robust hyperplane in the neighborhood of the sampled point $p_{i}$, and to then estimate the normal vector with respect to the hyperplane. We minimize the sum of Euclidean distances of the orthogonal projections (height) of points $q_{j}\in N_{g}\left(p_{i}\right)$, with respect to the hyperplane (Equation (3)). $\tau_{i}$ is the height to be found, which minimized the orthogonal projections of each point $q_{j}$ to the hyperplane.

(4) $$\arg\;\underset{x}{\min}\underset{j\in J}{\sum }\|p_{j}-x\|$$

The estimation of the hyperplane shows robustness to large deviations of points $q_{j}$. The outliers are identified by the L1-median height filter, which penalizes points $q_{j}$ with high orthogonal projections or heights $h_{i}$ with respect to the hyperplane.

Consequently, points $q_{j}$ with considerable heights, $h_{i}$, are probably located passing through the sharp features; these points are possible outliers. As such, we propose an adaptive weighting strategy, which adaptively assigns the weight of each point as a function of the height. Thus, the weighting term ψ(.), adaptively encourages the reduction of the influence of points $q_{j}$ with high $h_{i}$ values, and $\sigma_{h}$ is the height parameter, which controls sensitivity to outliers. Thus, the term ψ(.) only considers points located in the same smooth region to estimate the normal vector.

The L1 norm regularization term $E_{reg}$ is defined as

(5) $$E_{reg}=\underset{n_{j}\in N_{g}\left(n_{i}\right)}{\sum }w_{i,j}\|n_{i}-n_{j}\|{}_{1}$$

where $w_{i,j}=e^{-{\left(\frac{1-n_{i}^{t}n_{j}}{1-\cos\left(\sigma_{n}\right)}\right)}^{2}}$, which is introduced as a measure of sparsity to preserve the sharp features and smooth the underlying surface. If a point cloud is piecewise smooth, many of the gradients in the normals field n (consistently oriented) tend to be zero; in contrast, the large values of the gradient only indicate sharp features. This means that normals $n_{j}\in N_{g}(n_{i})$ in a neighborhood must be similar, where $w_{i,j}$ is the normal weight function, and $\sigma_{n}$ is the angle parameter that measures the similarity between normals $n_{j}$, which is customarily is set to $\sigma_{n}=15^{0}$.

### 3.4. Model Optimization

We optimize $n_{i}$ and $\tau_{i}$ subject to $\|n_{i}\|=1$, using a minimizing strategy defined as follows:

(6) $$\underset{n_{i},\tau }{\min}\sum_{q_{j}\in N_{g}\left(p_{i}\right)}\|h_{i}-\tau_{i}\|\psi \left(h_{i},\tau_{i}\right)\theta \left(\left\|p_{i}-q_{j}\right\|\right)+\lambda \sum_{n_{j}\in N_{g}\left(n_{i}\right)}w_{i,j}\|n_{i}-n_{j}\|{}_{1}.$$

This approach finds the optimal values of $n_{i}$ and $\tau_{i}$ by alternating optimization strategies, a procedure shown in Algorithm 1.

**Algorithm 1.** Model optimization1Initialization: $\tau_{0}=0$
2
*repeat*

 j=0
 *repeat*3  fix $\tau_{k}$, solve for $n_{k+1}$ as minimum of Equation (6).4  fix $n_{k}$, solve for $\tau_{k+1}$ as minimum of Equation (6).
  $p_{k+1}=p_{k}+\tau_{k}n_{k}$5 *until*
$\|p_{k+1}-p_{k}{\|}_{2}^{2}<\varepsilon$

 *edge_points_correction()*14
*until*
$j>j_{max}$


We solve the energy minimization problem regarding n having fixed τ. Since the minimization problem is non-differentiable due to the regularization term $E_{reg}$, we use the proximal gradient descendent method [33] as an optimization strategy.

#### 3.4.1. The $n_{0}$ Parameter Initialization n

First, we estimate the initial normal set to each $p_{i}\in P$ using only the equation corresponding to the fidelity term $E_{f}$, with τ=0 for the optimization. Similar to [34], we use the constraint ‖n‖=1, and compose the Lagrange form of Equation (3) to compute the derivative with respect to n, obtaining

(7) $$L\left(n,\lambda \right)=\underset{q_{j}\in N_{g}\left(p_{i}\right)}{\sum }\|h_{i}\|\psi \left(h_{i}\right)\theta \left(\left\|p_{i}-q_{j}\right\|\right)+\frac{\lambda }{2}\left(1-\|n_{i}\|{}^{2}\right)$$

(8) $$L_{n}\left(n,\lambda \right)=\underset{q_{j}\in N_{g}\left(p_{i}\right)}{\sum }\omega_{i}\left(p_{i}-q_{j}\right){\left(p_{i}-q_{j}\right)}^{t}n_{i}-\lambda n_{i}=0$$

with weight $\omega_{i}=\psi \left(h_{i}\right)\theta \left(\left\|p_{i}-q_{j}\right\|\right)/\left\|h_{i}\right\|$

We can see that the term $\omega_{i}\left(p_{i}-q_{j}\right){\left(p_{i}-q_{j}\right)}^{t}$ on Equation (8) is a symmetric and definite positive matrix (weighted covariance matrix), and we can rewrite it depending on n as

(9) Cm(n)n=λn

where $Cm\left(n\right)=\sum_{q_{j}\in N_{g}\left(p_{i}\right)}\omega \left(p_{i}-q_{j}\right){\left(p_{i}-q_{j}\right)}^{t}$

Equation (6) is an eigensystem and can be solved iteratively as follows:

(10) $$Cm\left(n_{k}\right)n_{k+1}=\lambda_{k+1}n_{k+1}$$

where $\lambda_{k+1}$ is the smallest eigenvalue of $Cm\left(n_{k}\right),$ and $n_{k+1}$ is an orthonormal eigenvector. We start the initialization with $n_{0}=0$, i.e., $Cm\left(0\right)n_{1}=\lambda_{1}n_{1}$, is the first iteration.

#### 3.4.2. Optimization of n

Keeping τ and $\psi \left(h_{i},\tau_{i}\right)$ fixed to solve Equation (6), $\psi \left(h_{i},\tau_{i}\right)$ is treated as a constant because it is a practical way to make it computationally tractable. Thus, the fidelity term $E_{f}$ has gradient $\nabla E_{f}$ as follows:

(11) $$\nabla E_{f}\left(n\right)=\sum_{q_{j}\in N_{g}\left(p_{i}\right)}\eta_{i}\left(h_{i}-\tau_{i}\right){\left(p_{i}-q_{j}\right)}^{t}$$

with weight $\eta_{i}=\psi \left(h_{i},\tau_{i}\right)\theta \left(\left\|p_{i}-q_{j}\right\|\right)/\left\|h_{i}-\tau_{i}\right\|$. $\eta_{i}$ is undefined when $h_{i}=\tau_{i}$; therefore, when $\left\|h_{i}-\tau_{i}\right\|<10^{-3}$, we set $\eta_{i}=\theta \left(\left\|p_{i}-q_{j}\right\|\right)$.

Setting $d=n_{i}-n_{j}$, we define the proximal mapping (or operator), associated with a convex non-differentiable function h(), as follows:

(12) $$prox_{h,\gamma }\left(d\right)=\underset{z}{\arg\;\min}\left(h\left(z\right)+\frac{1}{2\gamma }\|z-d{\|}_{2}^{2}\right)$$

The proximal gradient descendent has an iteration form as follows:

(13) $$d^{k+1}=prox_{h,\gamma }\left(d^{k}-\gamma \nabla E_{f}\left(n^{k}\right)\right)$$

where γ>0 is a scalar termed step size, and $d^{k+1}$ is computed iteratively until convergence. The proximal operator corresponding to the L1-norm or regularization term $E_{reg}$ is the following shrinkage or soft thresholding function:

(14) $$prox_{h,\gamma }\left(d_{ic}\right)=\{\begin{array}{cc} d_{ic}-\gamma \lambda w_{ij} & \mathrm{if}\;d_{ic}>\gamma \lambda w_{ij}\\ 0 & \mathrm{if}\;\left|d_{ic}\right|\leq \gamma \lambda w_{ij}\\ d_{ic}+\gamma \lambda w_{ij} & \mathrm{if}\;d_{ic}<-\gamma \lambda w_{ij} \end{array}$$

where $d_{ic}$ is each component of normal vector $d_{i}$.

#### 3.4.3. Optimization of τ

With n fixed, we solve Equation (6) for τ, which shows that the fidelity term $E_{f}$ has gradient $\nabla E_{f}$.

(15) $$\nabla E_{f}\left(\tau \right)=\underset{q_{j}\in N_{g}\left(p_{i}\right)}{\sum }\eta_{i}\left(h_{i}-\tau_{i}\right)=0$$

By solving $\nabla E_{f}\left(\tau \right)$, we obtain an iterative solution, which yields the following local update equation:

(16) $$\tau_{i}^{k+1}=\frac{\sum_{q_{j}\in N_{g}\left(p_{i}\right)}\eta_{i}h_{i}}{\sum_{q_{j}\in N_{g}\left(p_{i}\right)}\eta_{i}}$$

where $\eta_{i}=\frac{\psi \left(h_{i},\tau_{i}\right)\theta \left(\left\|p_{i}-q_{j}\right\|\right)}{\left\|h_{i}-\tau_{i}\right\|}$. The parameters n and τ are iteratively optimized using Equations (13) and (16) until convergence; $\eta_{i}$ is undefined when $h_{i}=\tau_{i}$. Therefore, when $\left\|h_{i}-\tau_{i}\right\|<10^{-3}$, we set $\eta_{i}=\theta \left(\left\|p_{i}-q_{j}\right\|\right)$.

### 3.5. Point Position Update and Point Border Correction

In the last stage of the denoising method, we follow the update vertex position with a distance-based constraint proposed by [25], where the resulted point cloud P is bounded within a prescribed distance to the input point cloud.

#### 3.5.1. Point Position Update

The authors in [25] propose a parameter provided by the user $\varepsilon \in R^{+}$, bounding the maximum deviation $d_{i}$ between the initial noisy point cloud and its corresponding iteratively denoised version point ${p^{\prime }}_{i}\in P^{\prime }$. The update position point ${p^{\prime }}_{i}$ for our algorithm is determined as follows:

(17) $${p^{\prime }}_{i}=\{\begin{array}{cc} p_{i}+\tau_{i}n_{i} & if\;d_{i}\leq \varepsilon \\ p_{i} & if\;d_{i}\geq \varepsilon \end{array},$$

where $d_{i}$ is computed as the difference between $p_{i}$ and the corresponding original point in the noisy point cloud. The parameter ε is set to 4h, i.e., ε=4h.

To make our algorithm more robust against edge artifacts and blurring, we detect the edge and corner points, and it corrects its position to obtain cleaner and more defined borders. 

#### 3.5.2. Edge Points Detection

To detect sharp features in the point cloud, we refer to the method proposed in [22], which uses the normals associated to each point in P and measures the normal variability into the neighborhood; if the variability is lower than a predefined threshold, th is labeled as edge point. The similarity between normal vectors $n_{i}$ and $n_{j}$ is defined as follows:

(18) $$w_{n}\left(n_{i},n_{j}\right)=exp\left(-\frac{\|n_{i}-n_{j}\|{}^{2}}{2\sigma_{n}^{2}}\right)$$

where $\sigma_{n}$ is an angle threshold; using Equation (18), we define the normal variation in $N_{g}(n_{i})$ as follows:

(19) $$V_{n}\left(i\right)=\frac{1}{\left|N_{g}(n_{i})\right|}\sum_{j\in N_{g}(n_{i})}w_{n}\left(n_{i},n_{j}\right).$$

All points that satisfy $V_{n}\left(i\right)<th$ are labeled as edge points.

#### 3.5.3. Edge Point Correction

**Figure 2:**
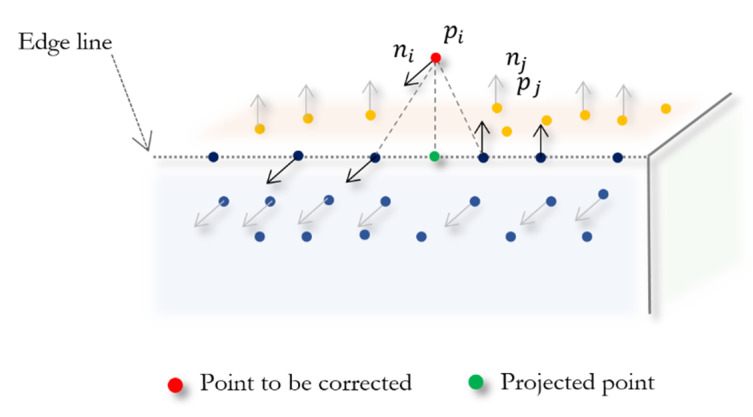
Edge points correction.

After edge points detection and taking advantage of the fact that the estimated normals near the edges and corners belong to surfaces on one side or another of the sharp features, we propose a scheme to correct the position of points that belong to edges or corners, which present a deviation from the corner or borderline. As shown in Figure 2, we find the closest point $p_{j}$ with normal vector $n_{j}$ on an opposite surface to the edge point $p_{i}$ and its normal $n_{i}$. Next, we project point $p_{i}$ onto the plane that contains point $p_{j}$ and its normal $n_{j}$. The new position is computed as

(20) $$d_{proj}=n_{j}^{\prime }\left(p_{i}-p_{j}\right).$$

We only correct the point positions that meet the following criteria:

(21) $$p_{corr}=\{\begin{array}{cc} p_{i}-d_{proj}\cdot n_{j}^{\prime } & if\;\delta <\left|d_{proj}\right|<\rho \cdot h\\ p_{i} & other\;case \end{array}$$

where h is the average distance between the points of the point cloud, ρ is a fixed value that represents the percentage of the maximum shift of point $p_{i}$ towards the edge line, and δ is a fixed value close to zero.

## 4. Experimental Results and Discussion

The proposed method was implemented in MATLAB and run on a laptop with Intel Core i7-2670QM CPU, 2.20 GHz processor, and 8 GB RAM. We tested the method using several point clouds with sharp features and smooth surfaces including irregular sampling. Additionally, synthetic and real scanned noisy point clouds were used to validate our method. The synthetic models were contaminated with Gaussian noise and impulsive noise along the normal directions or random directions. Different levels of Gaussian noise with zero mean and standard deviation σ were applied to the models; the standard deviation was proportional to the average distance between the points of the ground-truth point clouds. The noise of raw scanned data was natural. We compared our method with eight state-of-the-art denoising approaches including two MLS-based methods, APSS [8] and RIMLS [9]; one LOP based method, EAR [12]; one sparsity-based method, MRPCA [3]; one graph-based method, GLR [19]; and three normal smoothing-based methods, CNV [25], RN [24], and GN [22]. Methods APSS and RIMLS were implemented in MeshLab software. The GLR code and EAR software were provided by the authors, as were the results of the MRPCA, CNV, RN, and GN methods.

### 4.1. Parameter Selection and Tuning

Our method presented seven parameters: the sparsity parameter λ, the height sensitivity $\sigma_{h}$, the distance action range $\sigma_{d}$, the bound displacement ρ, the low bound δ, the radius of neighborhood r, and the total number of iterations k. 

The sparsity regularization parameter λ, depends on the desired gradient sparsity level and affects the reconstruction of sharp features and the smoothness of the point cloud. A larger λ yields a smoother result. We observed that λ=0.2 worked well for all the testing point sets used in the experiments.

The displacement ρ was fixed throughout all experiments with ρ=0.7. Parameter δ was fixed throughout all experiments with $\delta =10^{-4}$. Parameter h was the average distance between the points. We computed the value of h, taking the six nearest neighbors to each point. The distance action range $\sigma_{d}$, and the height sensitivity $\sigma_{h}$, are user-defined values given in terms of h. In all the experiments, the radius r of the neighborhood was set to $\sigma_{d}$, i.e., $r=\sigma_{d}$; a smaller value of $\sigma_{d}$ leads to faster computation because the neighborhood $N_{g}\left(p_{i}\right)$ is small, and large values may cross sharp features and over smooth the results. Alternatively, $r=\sigma_{d}$ can be chosen as a function of the local point density. In the results of the experiments, we chose the values of this parameter constant, tuned to achieve visually appealing results. The height sensitivity, $\sigma_{h}$, controls the outliers in the point cloud; small values of $\sigma_{h}$ preserve the model features, while large values only preserve the salient features.

The values of $\sigma_{h}$ and $\sigma_{d}$ depend on the level of noise. The bigger the noise level, the larger the value of these parameters that should be chosen. We used $\sigma_{d}\in \left\{1.5h,2h,3h,4h\right\}$ and $\sigma_{h}\in \left\{0.5h,0.7h,0.9h\right\}$ for synthetic data and $\sigma_{d}\in \left\{1.5h,2h\right\}$ and $\sigma_{h}\in \left\{0.1h,0.2h,0.3h\right\}$ for real scanned point clouds. The difference of parameter values between synthetic and real models was because the level of noise in real models was lower than synthetic models. The number of iterations k for the best results was set at k∈{10,16,20,50}. At last, there were only three parameters for our algorithm to tune the results $\left(\sigma_{h},\sigma_{d},k\right)$.

In our comparison experiments, we used the following parameter set for the eight selected state-of-the-art methods. For the methods [3,12,22,24], we mention Default in the parameter Table 1, because the corresponding smooth models were provided by the authors in [8]; we reported a tuple (scale, # of iterations, α); [9] = ($\sigma_{r},\sigma_{n}$); [12] = (Default values); [25] = (τ,ρ,p), and [19], the parameter settings in their paper. Our method = ($\sigma_{h},\sigma_{d},k$).

### 4.2. Quantitative Analysis

We used the following three metrics in our quantitative analysis: feature preservation, accuracy, and signal-to-noise ratio. 

#### 4.2.1. Error Metrics

To quantify feature preservation, we measured the orientation error between the smoothed point cloud and the ground truth. Mean angular deviation (MAD) was defined to measure the orientation error as follows:

(22) $$MAD=\frac{1}{n}\overset{n-1}{\underset{i=0}{\sum }}<\left({\bar{n}}_{i},{\hat{n}}_{i}\right)$$

where ${\bar{n}}_{i}$ and ${\hat{n}}_{i}$ are the point normals corresponding to the ground truth and the smoothed point cloud, respectively. 

To quantify the accuracy, i.e., the closeness between the ground truth model and the smoothing model, we used the mean-squared-error (MSE) metric, which measures the average of the squared Euclidean distances between the ground truth points and their closest denoising points, and vice versa between the denoised points and their closest ground truth points, and finally, the average between two measures gives the MSE. If the ground truth model and the smoothed model are $P_{1}={\left\{p_{i}\right\}}_{i=1,.n_{1}}$ and $P_{2}={\left\{q_{j}\right\}}_{j=1,.n_{2}}$, the point clouds can be of different sizes, i.e., $n_{1}\neq n_{2}$. The MSE is defined as follows:

(23) $$MSE=\frac{1}{2n_{1}}\sum_{p_{i\in P_{1}}}\underset{q_{j\in P_{2}}}{\min}\|p_{i}-q_{j}\|{}_{2}^{2}+\frac{1}{2n_{2}}\sum_{q_{i\in P_{2}}}\underset{p_{j\in P_{1}}}{\min}\|q_{j}-p_{i}\|{}_{2}^{2}$$

Finally, the signal-to-noise ratio (SNR) is measured in dB and is defined as follows.

(24) SNR=10log1n2∑qi∈P2‖qi‖22MSE

#### 4.2.2. Metric Evaluation 

Table 2 and Table 3 shows the comparison between our method and competing state-of-the-art methods highlighting cells in gray indicating the best performance. In Table 2 we can also see that the MSE and SNR metrics are the lowest values compared to eight state-of-the-art methods. For the Fandisk model, the proposed method reached the second position in all three metrics. For the Rocker Arm, our method outperformed the state-of-the-art methods in the MAD metric, but with MSE and SNR our method was better than the APSS method. For the Octahedron model, our method outperformed all metrics of the state-of-the-art methods.

**Figure 3:**
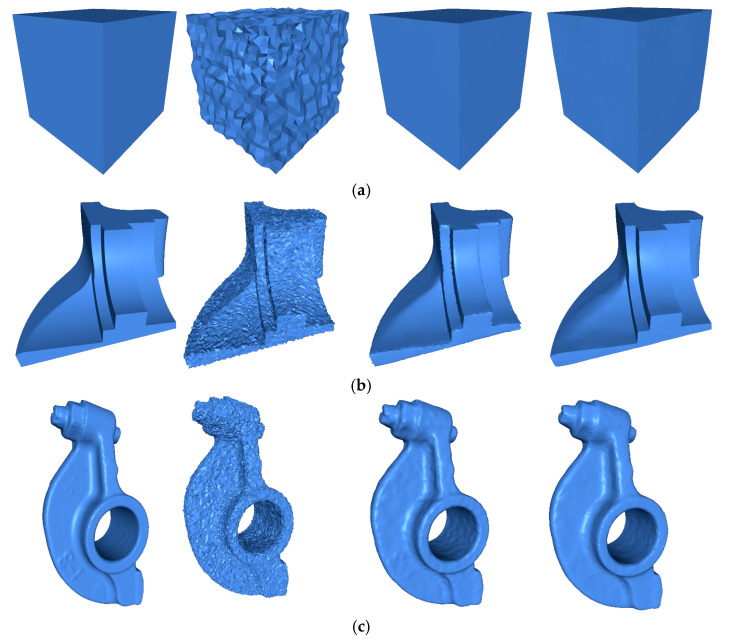

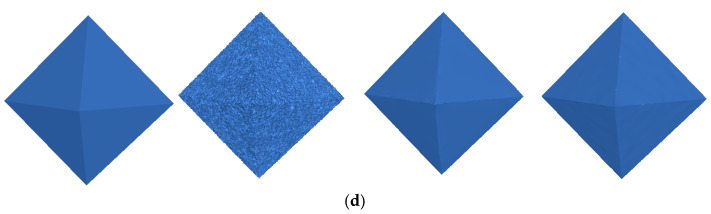
The first column shows the model from the original data. The second model is corrupted by Gaussian noise. The third column shows most accurate result of the comparison methods using MSE metric. The fourth column shows the result of correcting by the proposed method. The Gaussian noise levels and the comparison method are (**a**) Cube σ=0.3h method CVN, (**b**) Fandisk σ=0.28h method RN, (**c**) Rocker Arm σ=0.3h, and (**d**) Octhaedron σ=0.3h.

Table 3 shows a comparison between four state-of-the-art methods, with three different levels of noise, i.e., σ=0.1 h,σ=0.2 h, and σ=0.3 h. We can see that the MAD grew as the noise increased. Thus, if the noise level is high, the orientation error will be larger compared to the lower noise level. Table 3 shows that the proposed method achieved the best results for the MAD metric, with all levels of noise, but for the level of noise σ=0.1 h, RIMLS achieved the best MSE and SNR. However, for the level of noise σ=0.2 h and σ=0.3 h, our method outperformed the competing methods. In all the experiments, the proposed method achieved the best results on average in all three metrics and all noise levels. The visual results of the experiment are shown in Figure 3. For each one of the objects, we illustrated the original model, the noisy model, the results of the best method when comparing methods, and the results of our approach.

### 4.3. Qualitative Analysis

For visual comparison, we used the ball pivoting algorithm (BPA) [35] to reconstruct the mesh from the smoothed point cloud. The point clouds were contaminated with Gaussian noise along random directions and normal directions, and impulsive noise along random direction.

#### Irregular Point Clouds

**Figure 4:**
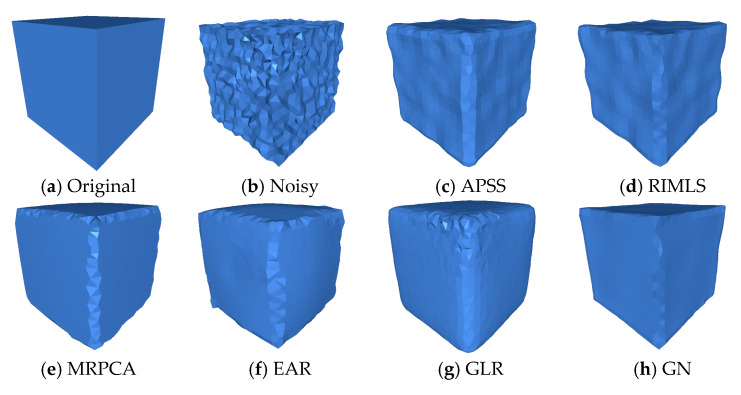

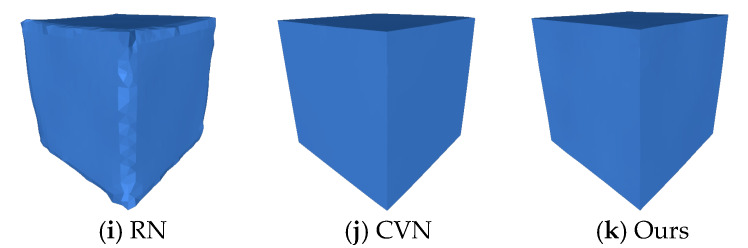
The Cube model with non-uniform distribution of points, corrupted by Gaussian noise (σ=0.3 h) along all directions, where h is the average distance between the points of the point cloud. We can see that the proposed method was able to preserve sharp features effectively when compared to the state-of-the-art methods. The surface was reconstructed using the ball pivoting algorithm.

**Figure 5:**
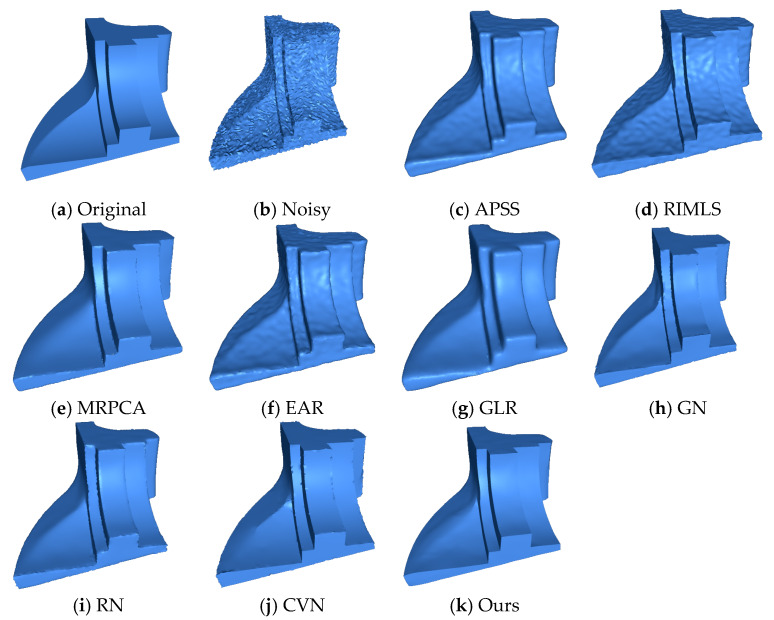
The Fandisk model with non-uniform distribution of points, corrupted by Gaussian noise (σ=0.28 h). We can see that the proposed method was able to preserve sharp features effectively when compared to the state-of-the-art methods.

The Cube and the Fandisk objects had non-uniform density points corrupted by Gaussian noise in random directions (σ=0.28 h,σ=0.3 h, and 0.3 h, respectively). Figure 4, shows that our method could preserve the sharp features and not produce bumps in flat areas like the APSS [8], RIMLS [9], and EAR [12] methods. GLR [19], MRPCA [3], RN [24], and GN [22] methods cleaned the noise effectively over flat regions but produced over-smoothing in the corners and edges. CNV [25] properly reconstructed the sharp features and cleaned the flat areas, but small artifacts appeared in some corners. As seen in Figure 5, our method could reconstruct sharp features and shallow features. APSS smoothed around the sharp features and did not remove the noise correctly. RIMLS and EAR preserved sharp features but produced some bump features in the resulting models. MRPCA removed the noise and preserved some sharp features but smoothed shallow areas around flat regions. GLR removed noise effectively but over smoothed the sharp features and shallow areas. GN, RN, and CVN produced a similar output to our method, but there were some artifacts on the borders and in corners.

### 4.4. Impulsive Noise

**Figure 6:**
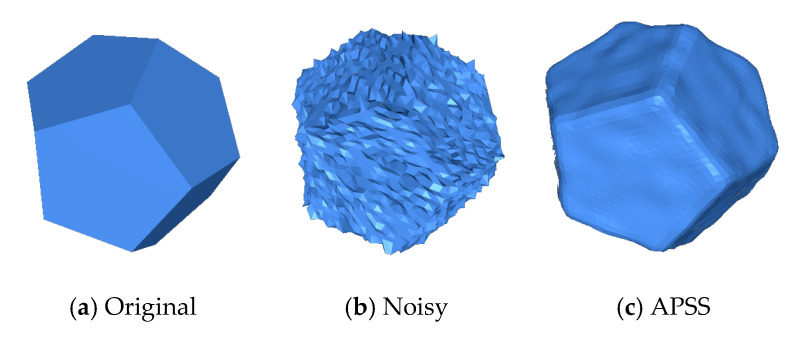

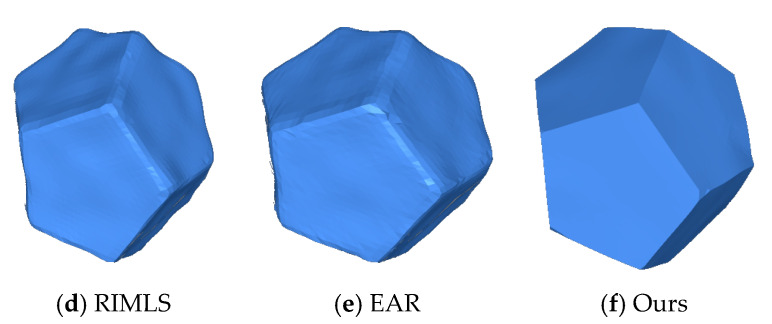
The Twelve model corrupted by impulsive noise ( σ=0.5 h) in the normal direction. We can see that the proposed method was able to preserve sharp features effectively when compared to the state-of-the-art methods. The surface was reconstructed using the ball pivoting algorithm.

Figure 6 shows the results of handling a corrupted point cloud adding an impulsive noise of σ=0.5 h along the normal direction. The Twelve model was smoothed by the proposed method and its edges were preserved. RIMLS, APSS, and EAR methods were not able to smooth the noise properly and reconstruct the edges.

### 4.5. Natural Noise of 3D Scan Objects

**Figure 7:**
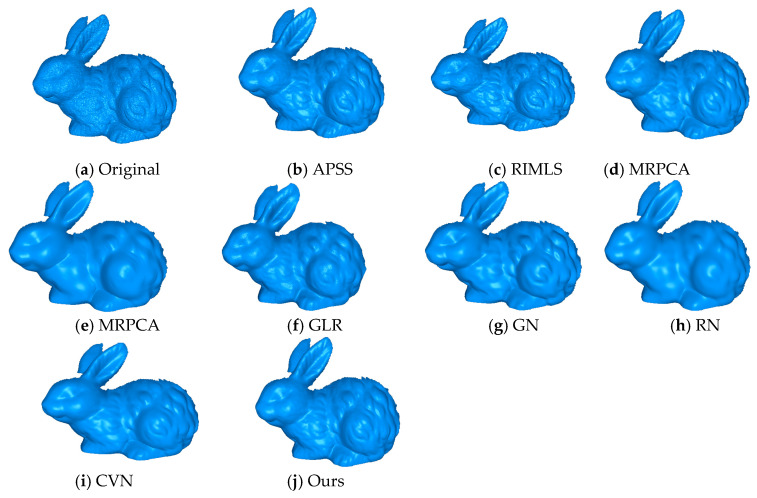
The rabbit model, with natural noise. We can see that the proposed method was able to preserve sharp features effectively when compared to the state-of-the-art methods. The surface was reconstructed using the ball pivoting algorithm.

We also compared these approaches using real scanned data of free form objects. Figure 7 shows the results of different methods applied to raw data scans. From the Rabbit object, it seems like our method effectively removed the noise while preserving features when compared to APSS, RIMLS, and EAR.

**Figure 8:**
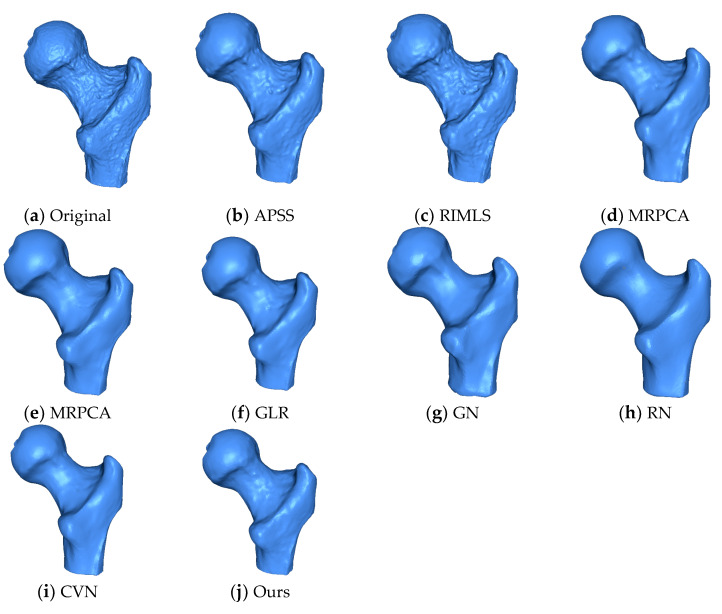
The ball joint model. We can see that the proposed method was able to preserve sharp features effectively when compared to the state-of-the-art methods. The surface was reconstructed using the ball pivoting algorithm.

GLR and RN preserved features, but lost some fine details as the eye and grooves in the ear were lost. GN and CVN preserved more detail than any of the other methods but they lost details in the eye and nose. MRPCA and the proposed method produced very similar results. Figure 8 shows the ball joint medical data. We can see that our method removed the noise, while details and sharp features were preserved, and the spherical shape was effectively smoothed. The APSS and RIMLS methods were not able to smooth the noise properly, and the resulting surfaces presented bumps. MRPCA, GN, RN, and GLR effectively removed the noise component but smoothed the sharp features. The EAR and CVN methods produced noise-free results, preserving the sharp features and smoothing the surfaces.

## 5. Conclusions

In this paper, we proposed combining the L1 median filter and the L1 norm regularization for a point cloud-based denoising algorithm that preserves sharp features. The algorithm uses double sparsity modeling both in the fitting term and in the regularization term. The L1 median is insensitive to outliers and noise, while the L1 norm preserves the sharp features and smooths the surface. The combined L1-median and L1-norm cost function was optimized with an alternating minimization strategy using a proximal gradient and a descendent iterative schema, allowing the implementation of a simple algorithm. The proposed method can handle models contaminated with Gaussian and impulse noise. High noise levels can produce erroneous results as they affect the normals estimation. Another issue is the concern with irregular point sampling models. While the irregular sampling remains low, the output of our algorithm produces good results; but when the point cloud density is highly irregular, the output quality decreases. To recover the sharp features, we introduce a border correction procedure that helps to correct edges and corners, preserving the models’ original sharp features.

Experimental results reveal that our proposal can preserve sharp features when compared to previous point cloud denoising methods, and the algorithm is robust in denoising both synthetic and raw point scans. The method depends on some empirical parameters, $\sigma_{h}$ and $\sigma_{d}$, defined by the user, and which we tuned manually to obtain the desired results. How to determine these parameters continues to be a challenge and is a direction we are going to investigate in our future research. The implementation of a global solution for the cost function is another issue to be examined in future work.

## Figures and Tables

**Table 1:** Parameter settings of comparative methods for different models.

Methods	Cube	Fandisk	Rocker Arm	Octahedron
EAR	Default	Default	Default	Default
APSS	(2, 45, 0.5)	(4, 15, 0)	(4, 15, 0.5)	(2, 45, 0.5)
RIMLS	(4, 0.75)	(4, 15, 0)	(4, 1)	(4, 0.75)
MRPCA	Default	Default	Default	Default
GLR	Paper	Paper	Paper	Paper
GN	Default	Default	Default	Default
RN	Default	Default	Default	Default
CNV	(0.3, 0.95, 150)	(0.3, 0.9, 150)	(0.25, 0.9, 80)	(0.25, 0.9, 80)
Ours	(0.98 h, 4 h, 30)	(0.7 h, 3 h, 16)	(0.7 h, 3 h, 14)	(0.7 h, 2 h, 20)

**Table 2:** Error metrics by comparative methods for each one of the 3D objects.

	Methods	EAR	APSS	RIMLS	MRPCA	GLR	GN	RN	CNV	Ours
Cube	MAD	4.3634	5.5588	4.4552	4.4341	6.2429	3.4878	4.4531	2.8668	2.6985
	MSE	0.0183	0.0125	0.0117	0.0273	0.0336	0.0330	0.0352	0.0064	0.0046
	SNR (dB)	39.438	42.613	43.272	35.749	33.936	33.920	33.305	48.394	51.418
Fandisk	MAD	4.4038	5.0465	5.6874	3.7932	7.7937	2.9186	3.1585	3.5273	2.9691
	MSE	0.0073	0.0057	0.0060	0.0067	0.0257	0.0060	0.0038	0.0108	0.0039
	SNR (dB)	45.105	46.168	45.965	45.525	39.653	45.963	48.006	43.410	47.833
Rocker Arm	MAD	5.9647	4.8825	4.9493	6.0163	7.1012	7.7694	5.7894	7.1894	4.2611
	MSE	0.1392	0.0468	0.0717	0.1345	0.2554	0.6141	0.5873	0.1651	0.0665
	SNR (dB)	36.116	40.830	38.988	36.234	33.450	29.717	29.885	35.340	39.300
Octahedron	MAD	1.8779	3.9838	4.8495	4.9541	5.2574	1.3606	1.3776	1.0415	1.0196
	MSE	9.5E4	0.0014	0.0011	0.0014	0.0016	0.0074	0.0074	7.0E4	5.6E4
	SNR (dB)	54.384	51.007	52.846	51.006	50.008	55.731	55.631	57.057	58.931

**Table 3:** The results of the error metrics of different compared methods for two Block and Trim-star objects varying the noise levels.

		Methods	EAR	APSS	RIMLS	GLR	Ours
σ=0.1h	Block	MAD	3.8083	4.2386	3.1723	2.9909	2.9232
		MSE	0.0693	0.0641	0.0466	0.0469	0.0477
		SNR(dB)	34.518	34.842	36.238	36.199	36.124
	Trim-star	MAD	5.0802	4.7813	4.1111	7.0203	3.6042
		MSE	0.0634	0.0324	0.0408	0.1105	0.0370
		SNR(dB)	29.572	32.459	31.472	27.120	31.855
σ=0.2h	Block	MAD	6.2682	8.2802	4.1979	4.9876	3.5737
		MSE	0.1339	0.1191	0.0688	0.0911	0.0551
		SNR(dB)	31.676	32.149	34.552	33.310	35.492
	Trim-star	MAD	6.9177	7.0610	5.3676	8.3487	4.8816
		MSE	0.1068	0.0525	0.0573	0.1456	0.0522
		SNR(dB)	27.341	30.353	30.011	25.914	30.363
σ=0.3h	Block	MAD	7.6707	11.629	4.6145	9.2070	4.4352
		MSE	0.1573	0.1640	0.1029	0.2735	0.0784
		SNR(dB)	30.980	30.753	32.816	28.488	33.955
	Trim-star	MAD	8.1821	10.9294	6.7664	10.495	5.8392
		MSE	0.0695	0.0995	0.0822	0.1828	0.0632
		SNR(dB)	29.158	27.576	28.465	24.915	29.608

Noise levels and the comparison method are (a) Cube σ=0.3 h method CVN, (b) Fandisk σ=0.28 h method RN, (c) Rocker Arm σ=0.3 h, and (d) Octhaedron σ=0.3 h.
